# Effect of extraction methods on the efficiency of sumac (*Rhus coriaria* L.) fruit extract in soybean oil quality during accelerated conditions

**DOI:** 10.1002/fsn3.2919

**Published:** 2022-05-12

**Authors:** Sepideh Rahmati, Behnaz Bazargani‐Gilani, Narjes Aghajani

**Affiliations:** ^1^ Department of Food Hygiene and Quality Control Faculty of Veterinary Science Bu‐Ali Sina University Hamedan Iran; ^2^ Department of Food Science and Technology Bahar Faculty of Food Science and Technology Bu‐Ali Sina University Hamedan Iran

**Keywords:** hydro‐ethanolic sumac extract, immersion method, oil oxidative stability, optimization, response surface methodology

## Abstract

Herbal extracts containing natural bioactive substances with numerous beneficial effects have been recently noticed as appropriate alternatives for synthetic food preservatives. In this study, we aimed to optimize the effects of different sumac (*Rhus coriaria*) fruit extracts (SFE) on oxidative stability of soybean oil under accelerated conditions compared to a synthetic antioxidant. Hydro‐ethanolic extracts (70%) of sumac fruits were prepared by three methods of immersion (I‐SFE), ultrasound (U‐SFE), and microwave (M‐SFE). According to the response surface methodology (RSM), 13 runs were considered in the concentrations of 0, 500, and 1000 ppm of each extract that were added to the soybean oil and stored at 60°C for a 20‐day period. All of the treatments were significantly (*p* < .05) efficient in preventing the chemical and sensory changes of soybean oil compared to the control in the dose‐dependent manner during storage period. I‐SFE treatment showed the lowest peroxide value (PV) (0.000063 meq (milliequivalents) O_2_/kg oil), thiobarbituric acid reactive substances (TBARS) (115.06 MDA (malondialdehyde)/kg oil), and acid value (0.0169 mg KOH (potassium hydroxide)/kg oil) among the other extracts at the end of the storage period. Furthermore, I‐SFE treatment earned the highest sensory scores (flavor, color, odor, and overall acceptability) of soybean oil in the range of 4–5 in comparison to the other treatments and synthetic antioxidant during storage time. According to the analysis of RSM, I‐SFE in the concentration of 999.998 ppm could optimally enhance the shelf life of soybean oil for 11.3614 days under accelerated conditions. It was concluded that I‐SFE with the same efficiency as synthetic antioxidants can be considered as a suitable alternative in soybean oil with various health benefits.

## Graphical abstract

It was concluded that immersion‐assisted extraction method‐sumac fruit extract (I‐SFE) with the same efficiency as synthetic antioxidants can be considered as a suitable alternative in soybean oil with various health benefits.

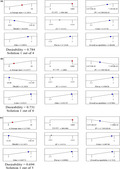

## 1 INTRODUCTION

Soybean is widely used for the manufacture and formulation of edible oil in many countries. Soybean oil is rich in polyunsaturated fatty acids (PUFA), disposed to the oxidation phenomenon and free radical production during storage period or under heat conditions, such as frying and cooking. Lipid oxidation is one of the spoilage reactions in foods that can lead to the quality and customer‐friendliness loss. Food manufacturers are forced to use synthetic antioxidants, such as butylated hydroxytoluene (BHT) and butylated hydroxyanisole (BHA), to combat lipid oxidation phenomenon; but, they are not sure about the safety effects of these compounds on consumer health. These concerns always exist about the use of synthetic additives in food (Tinello & Lante, 2020; Umeda & Jorge, 2021). The recent research introduced natural replacements for solving this problem. Herbal extracts and essential oils with numerous beneficial effects can be appropriate choices (Bagheri et al., 2016; Javadian et al., 2017). Most of these plants are found in many areas of the world and in addition to antioxidant and therapeutic properties, they can improve the sensory features, such as taste, odor, color, and overall acceptability of the food. Therefore, these agro‐food products can be used for producing functional foods (Basturk et al., 2018; Tinello & Lante, 2020; Umeda & Jorge, 2021). Sumac plant with the scientific name of *Rhus coriaria* L., belonging to the Anacardiaceae family, grows widely from Mediterranean to Iran and Afghanistan. In many countries, sumac fruit powder is served with various foods as a pleasant and popular condiment. Previous studies have demonstrated antioxidant activities of sumac fruit extract in laboratory conditions and food models. They reported that sumac fruit extracts contain phenolic acids, flavonols, anthocyanins, hydrolyzable tannins, and organic acids, such as malic, citric, and tartaric acids (Alsamri et al., 2021).

Extraction method can significantly affect the release amount of the bioactive substances in the extracts. Therefore, depending on the extraction method, the produced extract will have different physicochemical properties (Geow et al., 2021). Immersion‐assisted extraction method is the traditional solid–liquid extraction technique, in which the sample is placed in contact with the solvent at room temperature for long time with shaking until the bioactive phytochemicals present in the sample particles are completely solubilized and then released in the solvent (Azwanida, 2015; Geow et al., 2021). Ultrasound and microwave‐assisted extraction methods are the novel extraction approaches for the extraction of the bioactive substances of the plants. Ultrasound‐assisted extraction method creates the acoustic cavitation phenomenon in the solvent by the ultrasonic wave transition, which ruptures the cell membranes and enhances the contact surface between the plant sample and solvent. Therefore, the solvent penetrates into the plant cells, followed by releasing the effective substances of the samples. The generated heat by the microwave‐assisted extraction method has a great role in the yield of the phenolic compound extractions. This can be correlated to the high adsorption of microwave heat by the plant cell water, which leads to increasing water vapor pressure inside them, followed by the destruction of the cell wall and release of the phenolic compounds into solvent (Chemat et al., 2020; Sinan et al., 2020). In this regard, the objective of this work was to optimize and evaluate the effects of the produced sumac fruit extracts using three methods of immersion, microwave, and ultrasound at various concentrations on chemical (peroxide value (PV), thiobarbituric acid reactive substances (TBARS), and acid value (AV)) and sensory (flavor, color, odor, and overall acceptability) features of soybean oil stored at 60°C for 20 days compared to the synthetic antioxidant.

## 2 MATERIALS AND METHODS

### 2.1 Materials

Butylated hydroxytoluene (BHT), malondialdehyde (MDA), and Tween 80 were obtained from Sigma‐Aldrich Chemie. Analytical‐grade methanol, ethanol, chloroform, butanol, hexane–isopropanol, ammonium thiocyanate, iron chloride (II) and (III), potassium hydroxide, phenolphthalein, disodium hydrogen phosphate (Na_2_HPO_4_), sodium dihydrogen phosphate (NaH_2_PO_4_), thiobarbituric acid (TBA), and 1,1,3,3‐tetraethoxypropane (TEP) were procured from Merck.

### 2.2 Preparation of SFE

Sumac fruits were purchased from the local markets in Hamadan. After grinding the samples in the grinder (Hardstone, United Kingdom), mixed aqueous ethanol (70%) solvent with the ratio of 1:10 was obtained and then extracted by immersion (I‐SFE), ultrasound (U‐SFE), and microwave (M‐SFE) methods. In the immersion procedure, the samples were shaken in 250 rpm (revolutions per minute) for 24 hr. An ultrasound apparatus (FAPAN, Tehran, Iran) was used in the ultrasound method with the frequency of 20 kHz, the power of 50, and the temperature of 25°C for 30 min. In the microwave‐assisted extraction, the samples were extracted in a microwave oven (SolarDOM, LG, Seoul, Korea) with the frequency of 2450 MHz for 30 min. Next, the obtained solutions were filtered and concentrated by a rotary evaporator apparatus (Lab Tech) at 40ºC. Then, the remaining solvent was removed by vacuum oven at 50°C. After drying, the extracts were stored at −18ºC for subsequent uses (Albu et al., 2004; Barkhordari & Bazargani‐Gilani, 2021; Pan et al., 2008).

### 2.3 Preparation of the treatments

The fresh refined soybean oil, free of chemical antioxidants, was provided from an oil factory. The treatments were prepared in three groups, including: 1. SFE 0 ppm (soybean oil free of SFE), 2. SFE 500 ppm (soybean oil containing 500 ppm of SFE), and 3. SFE 1000 ppm (soybean oil containing 1000 ppm of SFE) for all three extracts. Tween 80 was used in 0.2% concentrations as an emulsifier. According to the design expert software, for these three groups, 13 runs were considered for all extracts separately that are defined in Table 1. Furthermore, soybean oil containing 200 ppm BHT was considered as the positive control. In other words, a total of 40 treatments were designated. All of the treatments were subjected to the accelerated test in an oven at 60 ± 1°C for 20 days and evaluated at 0, 10, and 20 days of storage period (Tinello & Lante, 2020; Umeda & Jorge, 2021).

**TABLE 1:** Designated treatments by design expert software analysis for each extraction method

Run	Block	Factor 1‐A: Storage time (day)	Factor 2‐B: SFE^a^ (ppm)
1	Block 1	10	500
2	Block 1	10	500
3	Block 1	0	500
4	Block 1	10	500
5	Block 1	20	1000
6	Block 1	20	0
7	Block 1	10	500
8	Block 1	0	1000
9	Block 1	10	500
10	Block 1	0	0
11	Block 1	10	0
12	Block 1	20	500
13	Block 1	10	1000

^a^ Sumac fruit extract.

### 2.4 Chemical analysis

#### 2.4.1 Peroxide value

Peroxide value (PV) was measured by the method suggested by the International Dairy Federation (IDF) (Shantha & Decker, 1994). PV was expressed as milliequivalents (meq) of O_2_ per kg of oil.

#### 2.4.2 Thiobarbituric acid reactive substances (TBARS)

The thiobarbituric acid reactive substances (TBARS) value was measured according to the Iranian National Standards (10,494–2006). A standard curve was determined using 1,1,3,3‐tetraethoxypropane (TEP) and the data were considered as milligrams (mg) of malondialdehyde (MDA) per kilogram (kg) of oil.

#### 2.4.3 Acid value

Acid values (AVs) of the samples were evaluated on the basis of the International Standard ISO (660–2009). AV was reported as milligrams (mg) of potassium hydroxide (KOH) per kilogram (kg) of oil.

### 2.5 Sensory analysis

A total of 28 undergraduate students were chosen from the Food Hygiene and Quality Control Department (20–22 years old) as the panelists for the sensory evaluation of the treatments. Fresh potatoes used for frying were purchased from the local markets. The potatoes (with dimensions of 7 × 0.3 × 0.5 cm) were peeled, washed, and sliced, using a stainless steel slicer. After adding salt (2%) to the potato slices, they were fried in the fryer (DF‐535T, Hamilton) in 180 ± 5°C for 5 min. The fried potato slices were placed and subjected to the panelists in plastic dishes for evaluating their sensory quality. A 5‐point Hedonic scale was considered to evaluate the color (1: Extremely undesirable, 5: Extremely great), odor (1: Extremely unacceptable/off‐odors, 5: Extremely pleasant), flavor (1: Extremely nonpalatable, 5: Extremely palatable), and overall acceptability (1: Extremely unacceptable, 5: Extremely pleasant) (Bazargani‐Gilani & Pajohi‐Alamoti, 2020; Ramos et al., 2012).

### 2.6 Statistical analysis

Response surface methodology (RSM) was used for the efficiency optimization of all three SFEs on the quality of stored soybean oil. For this purpose, central composite design in three levels and five repetitions in the central point were considered for the evaluation of SFE effects on the physicochemical and sensory characteristics of the heated soybean oil during storage time (+1, 0, −1) (Table 2). In this study, the independent variable limits, such as the concentrations of SFE and storage time, were found by the primary tests. Also, the obtained data were statistically analyzed by SPSS software (IBM SPSS statistics 21) and reported as mean values ±standard deviations (*SD*). The analysis of variance (ANOVA) and Tukey test were used at the significance level of *p* <.05 to compare the means.

**TABLE 2:** Applied independent variables along with limits and levels in the accelerated stored soybean oils for each extraction method

Independent variables	Levels and limits of variables		
	−1	0	+1
Storage time (day)	0	10	20
SFE^a^ concentration (ppm)	0	500	1000

^a^ Sumac fruit extract.

## 3 RESULTS AND DISCUSSION

### 3.1 Chemical analysis

#### 3.1.1 PV

**FIGURE 1:**
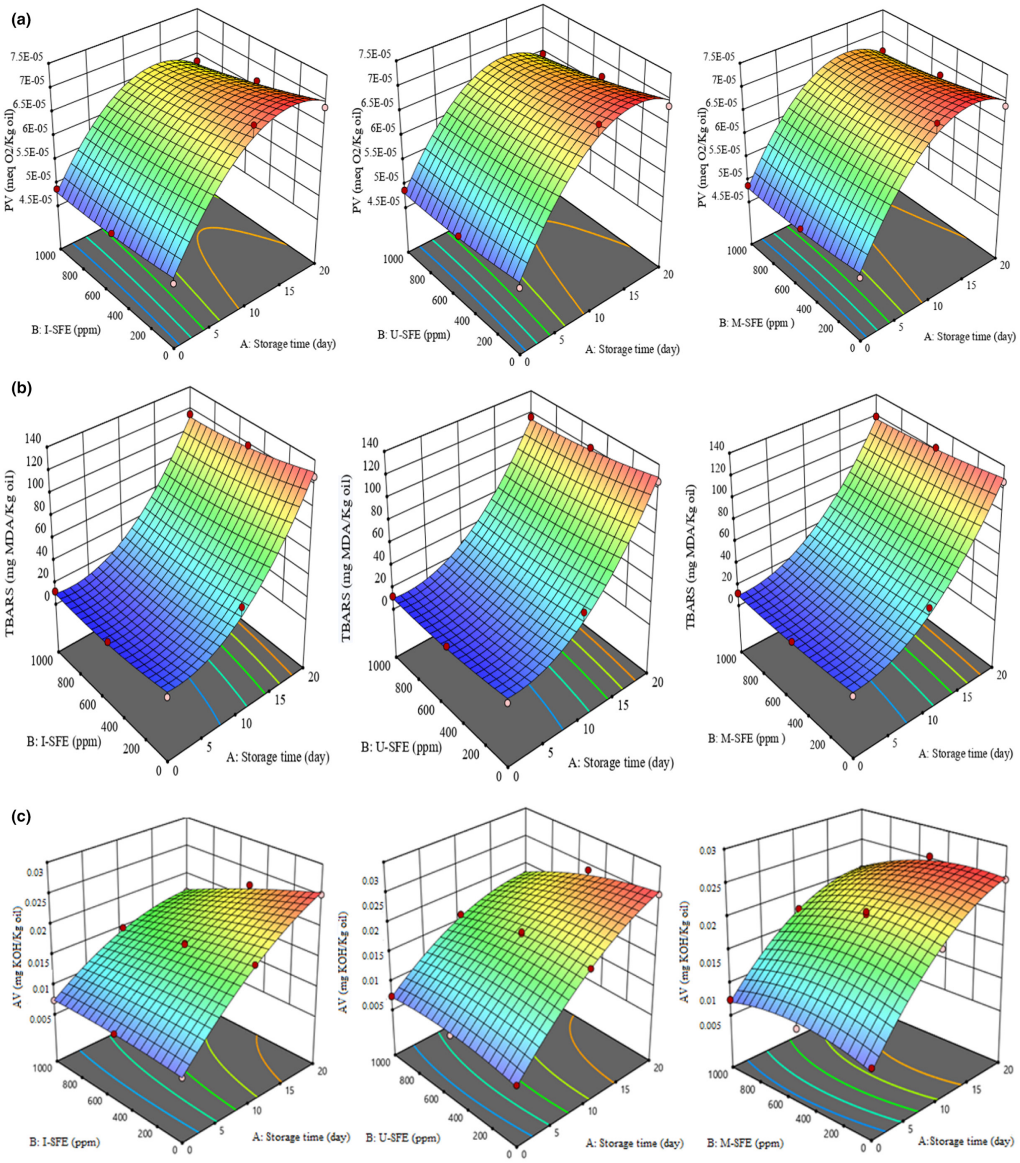
(a–c) Three‐dimensional (3D) diagrams of the changes in peroxide value (PV) (a), thiobarbituric acid reactive substances (TBARS) (b), and acid value (AV) (c) of the treated oils. I‐SFE, immersion‐assisted extraction method‐sumac fruit extract; U‐SFE, ultrasound‐assisted extraction method‐sumac fruit extract; and M‐SFE, microwave‐assisted extraction method‐sumac fruit extract

**FIGURE 2:**
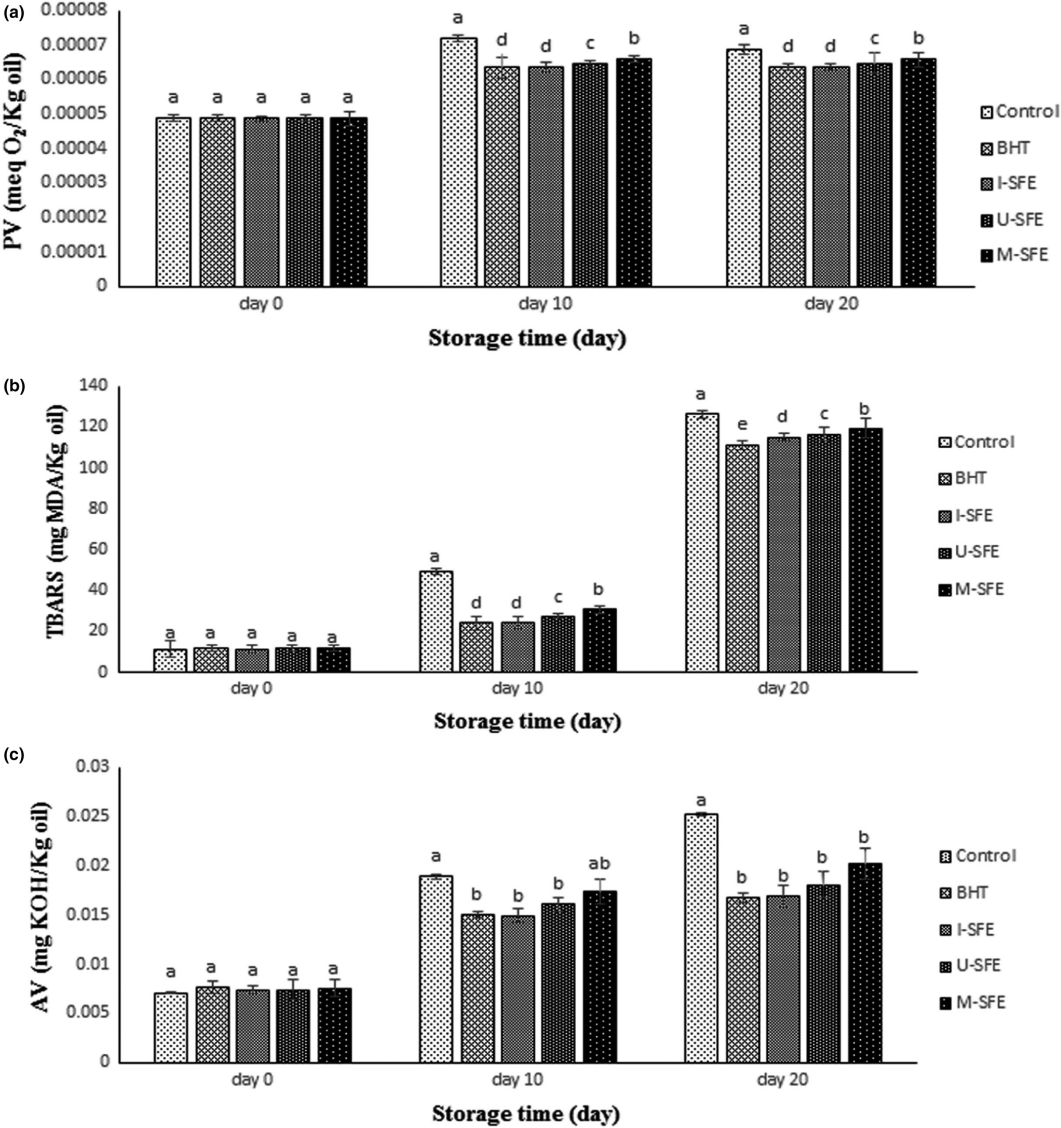
(a–c) Bar diagrams of the changes in peroxide value (PV) (a), thiobarbituric acid reactive substances (TBARS) (b), and acid value (AV) (c) of the treated oils during accelerated changes. I‐SFE (immersion‐assisted extraction method‐sumac fruit extract), U‐SFE, ultrasound‐assisted extraction method‐sumac fruit extract; and M‐SFE, microwave‐assisted extraction method‐sumac fruit extract. Different letters (a, b, c) indicate a statistically significant difference (*p* <.05)

The unstable free radical molecules made from triglycerides during oxidation reaction are called peroxides. Peroxides are tasteless and odorless molecules but decompose rapidly to hydro‐peroxides, followed by aldehyde compounds, which have strong unpleasant taste and odor. PV determines the produced peroxides as the primary products of oxidation reaction in the oils (Tinello & Lante, 2020). According to Figure 1a, by increasing the storage period of the treatments, an ascending trend of PV was observed until the 15th day. After that, a declining pattern appeared in PV until the end of the storage period. This can be correlated to decomposing unstable peroxides to secondary products of lipid oxidation, such as aldehydes during accelerated storage of the samples. Figure 2a–c illustrates chemical changes (a. PV, b. TBARS, and c. AV) of the studied oils containing the highest concentration of SFE (1000 ppm) in three extraction methods along with positive (BHT 200 ppm) and negative controls during 20 days of the accelerated storage. According to Figure 2a, no significant difference (*p* >.05) was observed in the PV of the samples in the initial day of the storage period. After that, the PVs of control, BHT, I‐SFE, U‐SFE, and M‐SFE treatments increased from 0.000048 to 0.000071, 0.000064, 0.000065, 0.000067, and 0.000069 meq peroxides/kg lipid after 10 days of storage and decreased thereafter to 0.000068, 0.000062, 0.000061, 0.000065, and 0.000067 after 20 days of storage, respectively. Therefore, I‐SFE and BHT treatments showed the lowest PVs among the others and M‐SFE, U‐SFE, and control groups were in the next ranks during the accelerated storage period. In other words, I‐SFE treatment exhibited the highest antioxidant activity compared to the others; this can be related to the increased release of the bioactive substances due to the increased extraction time in the immersion‐assisted extraction method.

Tinello and Lante (2020) reported an increasing trend in the PV of soybean oil during storage at 62°C for 28 days. They believed that high oxidative stability of the treated soybean oil containing ginger and turmeric powder compared to the control could be not only due to its higher phenolic yields and antioxidant activity but also due to the stability of their bioactive substances during storage under accelerated oxidation conditions. In another study, hydro‐ethanolic sumac extract (1000 ppm) showed better preventive effects on peroxide formation in corn oil compared to other treatments under accelerated oxidation at 60°C for 6 weeks. They reported that phenolic compounds, such as hydrolyzable tannins, anthocyanins, and the organic acids, such as malic and citric acids, are responsible for the antioxidant activity of sumac extract with different mechanisms in the heated corn oil (Baştürk et al., 2018). Umeda and Jorge (2021) reported that the natural antioxidant compounds in purple onion peel extract could preserve the quality of soybean oil up to the 7th day of accelerated storage in the oven at 60°C.

#### 3.1.2 TBARS

Figure 1b represents the three‐dimensional (3D) graphs of TBARS changes of the treated oils during accelerated storage. TBARS value measures malondialdehyde (MDA) as a secondary product in the termination phase of lipid oxidation reaction (Basturk et al., 2018). According to Figure 1b, by increasing the storage period, an ascending trend in the TBARS value of all studied samples was observed. In the first 10 days of the storage period, due to the peroxide formation in the initiation phase of lipid oxidation, the TBARS index showed low increase; while, in the second 10 days of the storage, by decomposition of peroxides to hydro‐peroxide and then more stable compounds, such as aldehyde and ketone products, the TBARS index exhibited the highest value for all treatments. Figure 2b illustrates statistical differences among the studied treatments during accelerated storage. The initial TBARS values of the studied groups were in the range of 11.45–11.89 mg MDA/kg oil and no significant (*p* >.05) difference was observed in them. In the control samples, the TBARS value increased from 11.72 to 126.16 mg MDA/kg oil, while TBARS values of BHT, I‐SFE, U‐SFE, and M‐SFE groups increased from 11.76 to 111.325, 11.45 to 115.06, 11.68 to 116.49, and 11.89 to 119.325 mg MDA/kg oil, respectively, after 20 days of storage. By increasing the storage time, BHT and I‐SFE groups significantly (*p* <.05) showed the lowest TBARS values among the other treatments and U‐SFE, M‐SFE, and the control were in the next ranks, respectively. The same order of the extract efficiencies in oxidation suppression was found in TBARS and PV tests. The inhibitory effects against the lipid oxidation of SFE followed the order of I‐SFE >U‐SFE >M‐SFE, in the descending trend.

In the microwave‐assisted extraction method, produced radiation waves penetrate the sample particles and change the electromagnetic energy into heat energy by two mechanisms of ionic conduction and dipole rotation. These mechanisms agitate the molecules and subsequently increase their temperature. Therefore, the heat‐sensitive bioactive compounds, such as tannins and anthocyanins, may be oxidized and degraded by this method (Gaber et al., 2018; Geow et al., 2021; Hu et al., 2017). The generated vibration in ultrasound‐assisted extraction method can create bubble formation in the solvent. By the overgrowth of the bubbles, they will collapse and produce cavitation effects. Due to the high‐energy release by the cavitation effect, microcracks are created on the solid particle surfaces. Therefore, the solvent can easily enter the sample particles through these microcracks and extract the bioactive substances. However, there are some limitations for the ultrasound‐assisted extraction method. The direct immersion of ultrasound probe in the sample will increase temperature in a short time during extraction process due to the low‐energy loss to the surrounding. Therefore, the heat‐sensitive substances can be damaged during extraction process by this method (Gaber et al., 2018; Geow et al., 2021; Perrier et al., 2017). Furthermore, the bioactive phytochemicals of the sample can be damaged by the ultrasound mechanical waves. Also, according to the previous reports, the use of ultrasound waves may have side effects on the bioactive phytochemicals through the generation of free radicals (Azwanida, 2015).

In the immersion‐assisted extraction method, by increasing the contact time of an appropriate solvent with the sample particles, the bioactive substances of the sample can be dissolved and released in the solvent (Gaber et al., 2018; Geow et al., 2021). The superiority of the simple immersion method to other techniques can be related to not using heat and mechanical waves during extraction process. Therefore, no damage threatens the sensitive bioactive compounds of the sample.

Basturk et al. (2018) reported a gradual increase of TBARS value in corn oil samples during accelerated storage. They observed that the inhibitory effects of sumac ethanolic extract on TBARS value were stronger than BHT, nettle seed, flax seed, sage, mint, and thyme treatments. They attributed this effect to the high phenolic content, followed by the significant antioxidant activities of sumac extract compared to other extracts and BHT. Also, they observed that sumac extract with 50% inhibition concentration (IC_50_) of 29.89 µg/ml exhibited a strong antioxidant activity in 1,1‐diphenyl‐2‐picryl hydrazyl (DPPH) radical scavenging assay. In another study, the TBARS value of sunflower oil significantly (*p* <.01) increased during accelerated storage. The authors reported that adding *Coriandrum sativum* essential oil to the sunflower oil could significantly (*p* <.05) decrease the TBARS index compared to the control in a dose‐dependent manner under the temperature of 65°C during 24 days of storage (Wang et al., 2018). Alsamri et al. (2021) reported that the sumac plant is rich in various classes of phytochemicals, including flavonoids, tannins, polyphenolic compounds, organic acids, and many others. According to the previous studies, the remarkable antioxidant activity of the sumac fruit ethanolic extract can be related to the high content of the bioactive substances such as gallicin, gallic acid, glucogallic acid, quercitrin, isohyperoside, myricetin glucuronide, tri‐galloyl‐hexoside, penta‐galloyl‐hexoside, myricetin rutinoside, dihydroxy‐methyl xanthone, β‐sitosterol‐hexoside, α‐tocopherol, and linoleic acid compounds (Abdallah et al., 2019). According to the reports of another study, sumac fruit extract and sumac‐synthesized nanoparticles exhibited reducing power and significant (*p* <.05) scavenging activity against DPPH and ABTS (2,2'‐azinobis‐(3‐ethylbenzothiazoline‐6‐sulfonic acid) free radicals (Bursal & Koksal, 2011; Ibrahim et al., 2019; Majd et al., 2017). Salimi et al. (2015) showed that the sumac fruit aqueous extract (50, 100, and 200 mg/kg) decreased malondialdehyde (MDA), a marker of oxidative stress, in the alloxan‐induced diabetic rats.

#### 3.1.3 AV

The AV (acid value) is milligram of the required potash to neutralize the free fatty acids in 1 gram of oil. This index measures the acidity content and therefore the quality of the oil (Mahesar et al., 2014). Figure 1c illustrates the AV changes of the samples during accelerated storage. By increasing the storage time, AV of all the treatments showed an ascending trend until the end of the storage period. In other words, the accelerated storage increased hydrolysis rate of triglycerides, followed by AV of the oil. According to Figure 2c, the initial (day 0) AV of soybean oil of all treatments was in the range of 0.0071–0.0076 mg KOH/kg oil. The control group significantly (*p* <.05) showed the highest AV (0.0189 and 0.0252 mg KOH/kg oil in day 10 and day 20 of the storage, respectively) among the other treatments. On the other hand, no significant difference (*p* >.05) was found among the studied treatments at the highest concentration (1000 ppm) of the extracts and positive control at the end of the storage time; while, consistent with the peroxide and TBARS values of the samples, the significant differences of AV were expected to be observed among the studied treatments during storage period (Wang et al., 2018). This may be correlated to the low accuracy of the titration method to find these differences. AV of the oils is measured by a titration method in an organic solvent, such as ethanol; then, potassium hydroxide (KOH) solution is used to titrate the acids in oil to a targeted pH end point. The presence of the organic solvent leads to a high mix of oil with the titrant, as a result of which the neutralization reaction will occur very quickly. Due to the weak and low content of fatty acids in the oils, finding a sharp pH end point in titration is unattainable. As a result, the measurement error for AV analysis will be inevitable. Therefore, a simple, efficient, and accurate method for the determination of acid value in edible oils has been recently recommended by a solvent‐assisted and reaction‐based headspace gas chromatography (HS‐GC), Fourier‐transform infrared (FTIR) spectroscopy, and spectrophotometric methods (Mahesar et al., 2014; Xie & Chai, 2016).

### 3.2 Sensory analysis

**FIGURE 3:**
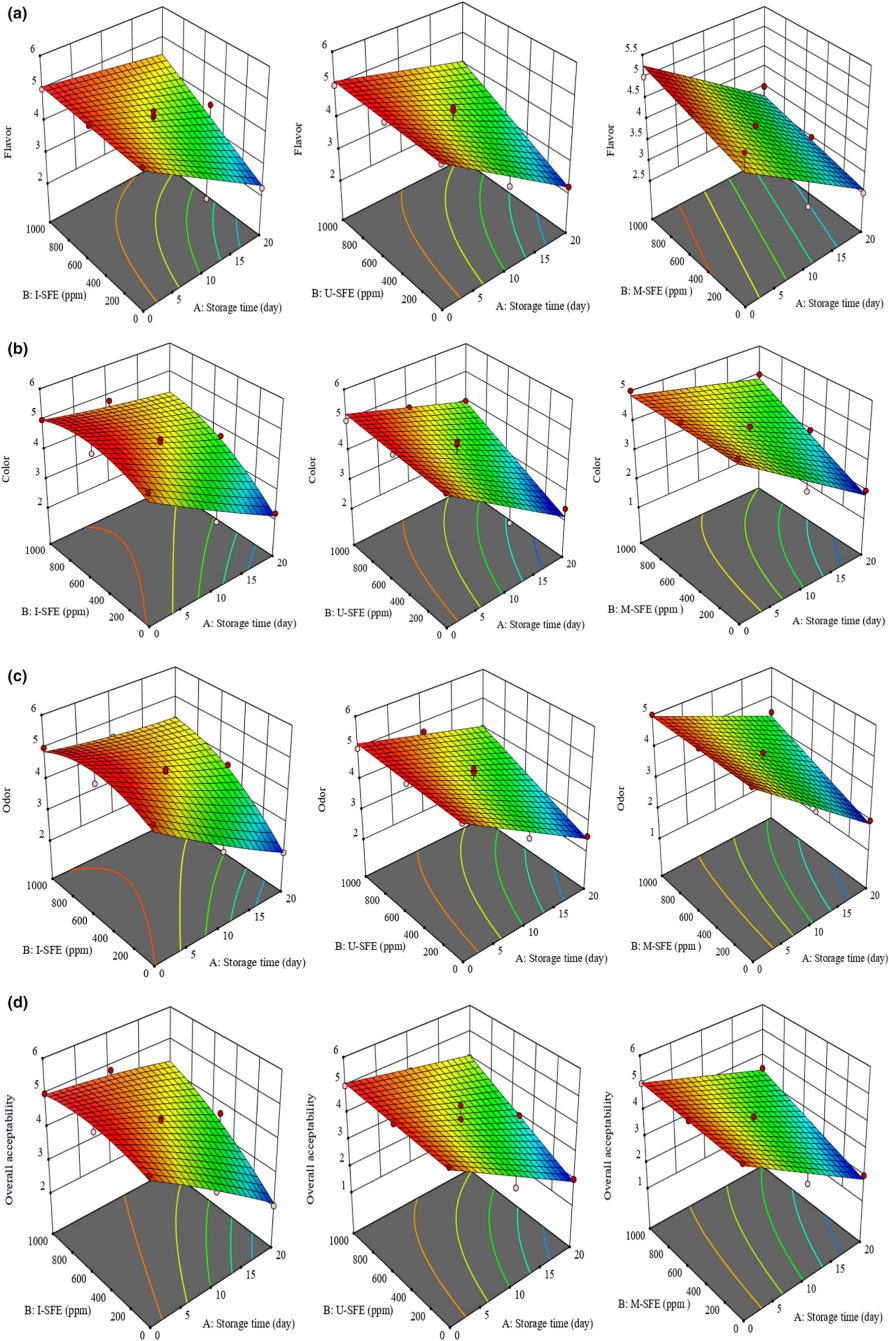
(a–d) Three‐dimensional (3D) diagrams of the changes in sensory features (flavor (a), color (b), odor (c), and overall acceptability (d)) of the treated oils during accelerated storage. I‐SFE (immersion‐assisted extraction method‐sumac fruit extract), U‐SFE, ultrasound‐assisted extraction method‐sumac fruit extract; and M‐SFE, microwave‐assisted extraction method‐sumac fruit extract

**FIGURE 4:**
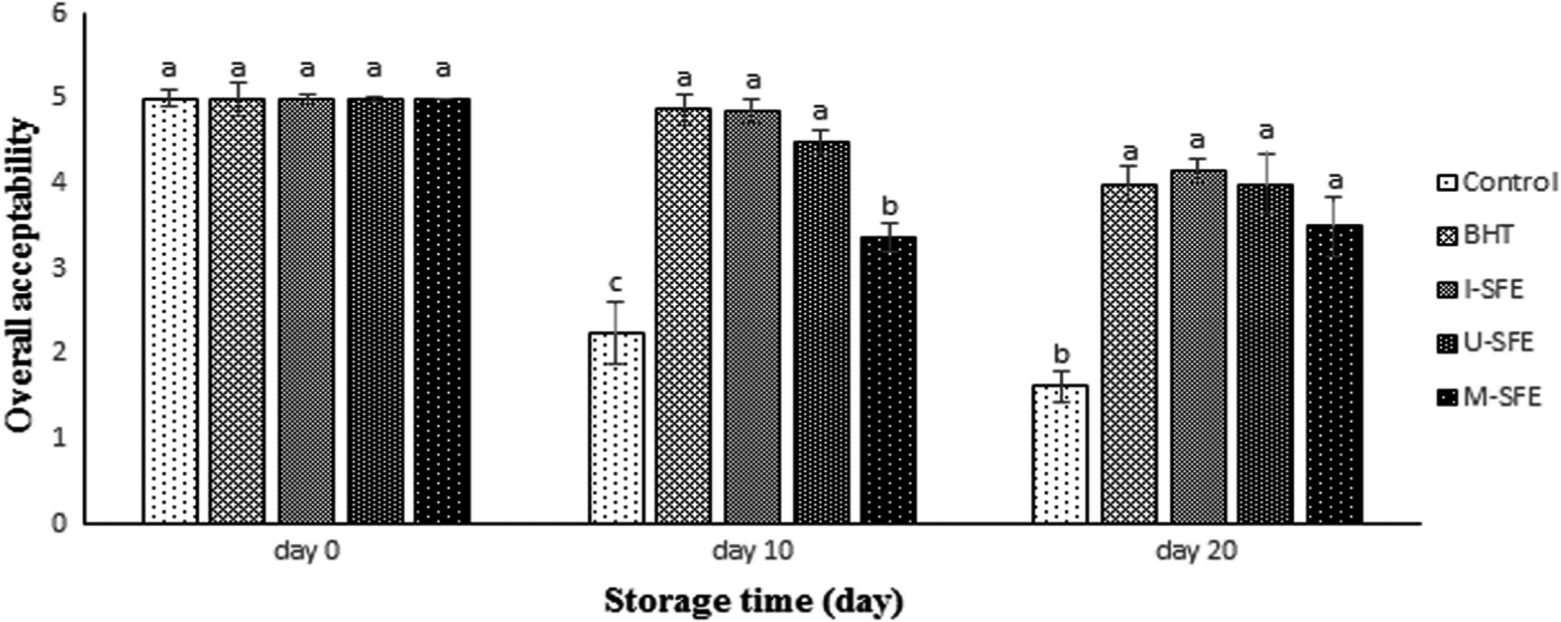
Bar diagrams of the changes in overall acceptability of the treated oils during accelerated storage. I‐SFE (immersion‐assisted extraction method‐sumac fruit extract), U‐SFE, ultrasound‐assisted extraction method‐sumac fruit extract; and M‐SFE, microwave‐assisted extraction method‐sumac fruit extract. Different letters (a, b, c) indicate a statistically significant difference (*p* <.05)

Figure 3a–d represents three‐dimensional (3D) graphs of flavor, color, odor, and overall acceptability changes of the fried potatoes in the treated soybean oils during accelerated storage period. According to the obtained findings, a descending trend was observed in all sensory features of the studied groups by increasing the storage time and conversely, an ascending trend was found in them by increasing the concentration of SFE; then, the highest sensory scores belonged to the SFE in the concentration of 1000 ppm during storage time. As shown in Figure 3a–d, all sensory scores of I‐SFE treatment were never lower than 4 in the highest concentration (1000 ppm) during accelerated storage time. Figure 4 represents statistical differences in the overall acceptability of the studied oil samples containing the highest concentration of SFE (1000 ppm) in three extraction methods along with positive (BHT 200 ppm) and negative controls during 20 days of the accelerated storage. Based on Figure 4, SFE‐containing treatments were significantly (*p* <.05) recognized as the most efficient treatments in the preservation of sensory features of the fried potatoes in soybean oil under accelerated conditions compared to the control group and exhibited the same efficiency as the synthetic antioxidant (BHT). The overall acceptability scores of control, BHT, I‐SFE, U‐SFE, and M‐SFE groups decreased from 5 to 1.625, 4, 4.15, 4, and 3.5 after 20 days of storage, respectively. These results were in agreement with chemical findings of the samples which may be due to the production of lipid oxidation products, such as peroxides and aldehydes, which may lead to the production of off‐odor and off‐flavor which may be a cause for the poor scores for these samples. Wang et al. (2018) reported that the addition of *Coriandrum sativum* essential oil to sunflower oil at 1200 ppm could improve its aroma, flavor, and consumers’ acceptability during accelerated storage period, so that it could be introduced as a convenient condiment. Nor et al. (2009) observed that the sensory features (color, flavor, oiliness, crispiness, taste, and overall quality) of the fried potatoes in palm olein containing *Curcuma longa* leaves extract significantly (*p* <.05) improved compared to the control and BHT treatments on the fifth day of frying. They reported that the fried potatoes in *Curcuma longa* leaf extract‐treated oil were acceptable up to the fifth day of frying. Meng et al. (2021) found a gradual descending trend in the sensory features of the sunflower oil during the entire storage period. They reported that the addition of Huai *Chrysanthemum morifolium* essential oil (HCEO) to the sunflower oil samples could improve the flavor, aroma, appearance, and overall acceptability scores of them during accelerated storage at 65*°*C for 30 days in a dose‐dependent manner. They concluded that HCEO could not only be used as a flavoring agent in sunflower oil, but could also be used to upgrade its sensory properties and oxidative stability during accelerated storage, which was in agreement with previous studies (Chandran et al., 2016; Wang et al., 2018).

### 3.3 Optimum storage conditions

**FIGURE 5:**
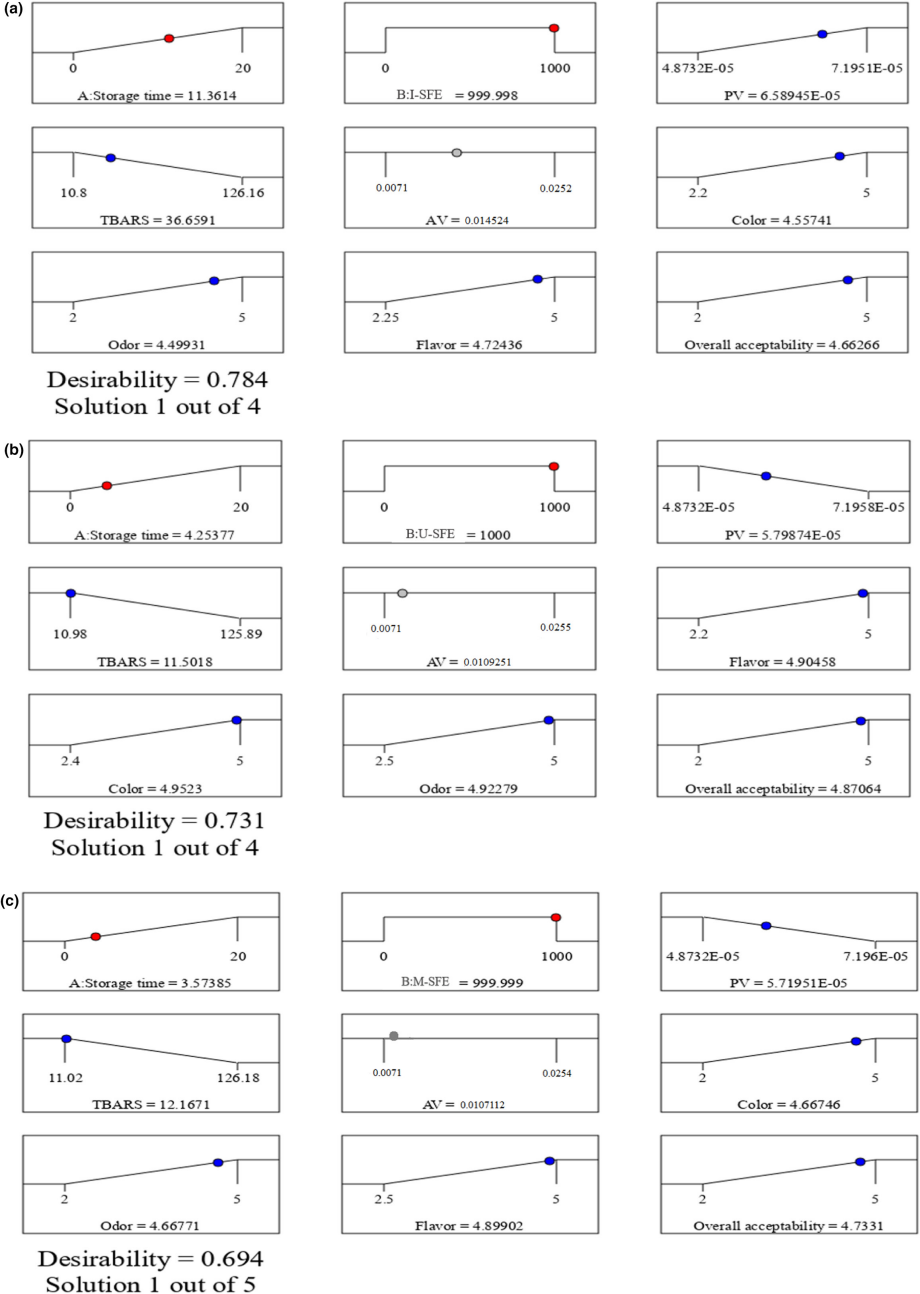
(a–c) The optimized storage conditions of the soybean oils containing I‐SFE (a), U‐SFE (b), and M‐SFE (c) under accelerated conditions. I‐SFE, immersion‐assisted extraction method‐sumac fruit extract; U‐SFE, ultrasound‐assisted extraction method‐sumac fruit extract; and M‐SFE, microwave‐assisted extraction method‐sumac fruit extract

According to the obtained findings and design expert software analysis (Table 3), the optimized storage conditions of the soybean oil were as follows: I‐SFE: 999.998 ppm for 11.3614 days, U‐SFE: 1000 ppm for 4.25377 days, and M‐SFE: 999.999 ppm for 3.57385 days (Figure 5a–c). In the above conditions, some of the dependent variables such as PV, TBARS, AV and others, such as sensory characteristics (flavor, color, odor, and overall acceptability), will be minimum and maximum, respectively. Based on our results, I‐SFE could optimally increase the shelf life of the soybean oil longer than other extracts under accelerated conditions.

**TABLE 3:** Considered limits for optimization of storage conditions of the soybean oils containing immersion‐assisted extraction method‐sumac fruit extract (I‐SFE), ultrasound‐assisted extraction method‐sumac fruit extract (U‐SFE), and microwave‐assisted extraction method‐sumac fruit extract (M‐SFE) under accelerated conditions

Conditions	Purpose	Minimum limit			Maximum limit		
		I‐SFE	U‐SFE	M‐SFE	I‐SFE	U‐SFE	M‐SFE
Storage time (day)	Maximum	0	0	0	20	20	20
Concentration (ppm)	In the range	0	0	0	1000	1000	1000
PV	Minimum	4.8732 × 10^–5^	4.8732 × 10^–5^	4.8732 × 10^–5^	7.1951 × 10^–5^	7.1958 × 10^–5^	7.196 × 10^–5^
TBARS	Minimum	10.8	10.98	11.02	126.16	125.89	126.18
AV	Minimum	0.0071	0.0071	0.0071	0.0252	0.0255	0.0254
Flavor	Maximum	2.25	2.2	2.5	5	5	5
Odor	Maximum	2	2.5	2	5	5	5
Color	Maximum	2.2	2.4	2	5	5	5
Overall acceptability	Maximum	2	2	2	5	5	5

Abbreviations: AV, acid value; PV, peroxide value; TBARS, thiobarbituric acid reactive substances.

## 4 CONCLUSION

It was concluded that SFE was effective in decreasing the chemical and sensory changes, followed by increasing the shelf life of the soybean oil during accelerated storage. This property of SFE was significantly affected by the kind of the extraction method, so that the immersion‐assisted extraction method was the most efficient technique in releasing the antioxidant substances in the solvent among the other methods and the ultrasound and microwave‐assisted extraction methods were in the next ranks, respectively. Furthermore, it was found that I‐SFE: 999.998 ppm for 11.3614 days, U‐SFE: 1000 ppm for 4.25377 days, and M‐SFE: 999.999 ppm for 3.57385 days are the optimum conditions for the shelf life enhancement of the soybean oil during accelerated storage; thus, I‐SFE with the same efficiency as the synthetic antioxidants can be introduced as a good alternative for them in the food preservation. Investigating SFE produced by other novel extraction methods with different solvents and concentrations can be proposed for food preservation in future studies.

## CONFLICT OF INTEREST

The authors declare that they have no known competing financial interests or personal relationships that could have appeared to influence the work reported in this paper.

## ETHICAL APPROVAL

This study does not involve any human or animal testing.

## Data Availability

Data available on request from the authors.
